# Bacterial flagellin promotes viral entry via an NF-kB and Toll Like Receptor 5 dependent pathway

**DOI:** 10.1038/s41598-019-44263-7

**Published:** 2019-05-27

**Authors:** Elizabeth K. Benedikz, Dalan Bailey, Charlotte N. L. Cook, Daniel Gonçalves-Carneiro, Michelle M. C. Buckner, Jessica M. A. Blair, Timothy J. Wells, Nicola F. Fletcher, Margaret Goodall, Adriana Flores-Langarica, Robert A. Kingsley, Jens Madsen, Jessica Teeling, Sebastian L. Johnston, Calman A. MacLennan, Peter Balfe, Ian R. Henderson, Laura J. V. Piddock, Adam F. Cunningham, Jane A. McKeating

**Affiliations:** 1 Institute of Immunology and Immunotherapy, University of Birmingham, Birmingham, UK; 2 Institute of Microbiology and Infection, University of Birmingham, Birmingham, UK; 3 The Pirbright Institute, Guildford, Surrey, UK; 4 Institute of Food Research, Norwich, UK; 5 Department of Child Health, University of Southampton, Southampton, UK; 6 Biological Sciences, University Hospital Southampton NHS Foundation Trust, Southampton, UK; 7 National Heart and Lung Institute, Imperial College London, London, UK; 8 Jenner Institute, Nuffield Department of Medicine, University of Oxford, Oxford, UK; 9 Nuffield Department of Medicine, University of Oxford, Oxford, UK

**Keywords:** Virology, Antimicrobial responses

## Abstract

Viruses and bacteria colonize hosts by invading epithelial barriers. Recent studies have shown that interactions between the microbiota, pathogens and the host can potentiate infection through poorly understood mechanisms. Here, we investigated whether diverse bacterial species could modulate virus internalization into host cells, often a rate-limiting step in establishing infections. Lentiviral pseudoviruses expressing influenza, measles, Ebola, Lassa or vesicular stomatitis virus envelope glycoproteins enabled us to study entry of viruses that exploit diverse internalization pathways. *Salmonella* Typhimurium, *Escherichia coli* and *Pseudomonas aeruginosa* significantly increased viral uptake, even at low bacterial frequencies. This did not require bacterial contact with or invasion of host cells. Studies determined that the bacterial antigen responsible for this pro-viral activity was the Toll-Like Receptor 5 (TLR5) agonist flagellin. Exposure to flagellin increased virus attachment to epithelial cells in a temperature-dependent manner via TLR5-dependent activation of NF-ΚB. Importantly, this phenotype was both long lasting and detectable at low multiplicities of infection. Flagellin is shed from bacteria and our studies uncover a new bystander role for this protein in regulating virus entry. This highlights a new aspect of viral-bacterial interplay with significant implications for our understanding of polymicrobial-associated pathogenesis.

## Introduction

Many viruses infect their host through mucosal surfaces that are coated with microbial life and recent advances show a dynamic interplay between bacterial and viral species and the host^1^. Several reports show that respiratory infections such as influenza or respiratory syncytial virus (RSV) induce epithelial cell damage that can potentiate bacterial colonization and secondary infections^2–5^. The bacterial encoded lipopeptide Pam3-Cys-Ser-Lys4 was reported to enhance RSV attachment to their target cells via an undefined mechanism^6^, highlighting a role for bacterial species to facilitate virus infection. Viral-bacterial co-infections are commonly observed in patients with cystic fibrosis or chronic obstructive pulmonary disease^7,8^ and in a caecal-ligation puncture model of sepsis, pulmonary murine cytomegalovirus virus infection was shown to reactivate and cause enhanced pathology^9^. Moreover, sepsis and bacterial meningitis is associated with reactivation of multiple virus types including Varicella Zoster and Herpes Simplex virus reactivation^10,11^ highlighting a role for polymicrobial infections to influence the development of chronic disease.

Viral entry into a host cell represents the first step in the infectious life cycle and is mediated via specific interactions between virus-encoded proteins and cellular receptors that define internalization pathways^12^. Polarized epithelia within the respiratory or gastrointestinal tracts act as barriers to restrict pathogen infection^13^. Many bacterial strains encode proteins, such as CagA from *Helicobacter pylori*, that promote their invasion by disrupting epithelial permeability^14^, providing a break in barrier function that could potentiate virus entry. Bacteria express a range of other molecules, such as the Gram-negative cell wall component lipopolysaccharide (LPS) or the motility protein flagellin that activate inflammatory responses^15,16^. Thus, there is significant scope for bacteria to alter host susceptibility to virus infection. However, to date there have been limited studies investigating the effect of such bacterial factors on virus infection. We designed a non-biased *in vitro* screen to investigate the impact of bacterial species on viral uptake into lung epithelial cells and to explore the underlying molecular pathways.

## Results

### Diverse bacterial strains promote virus entry into epithelial cells

**Figure 1:**
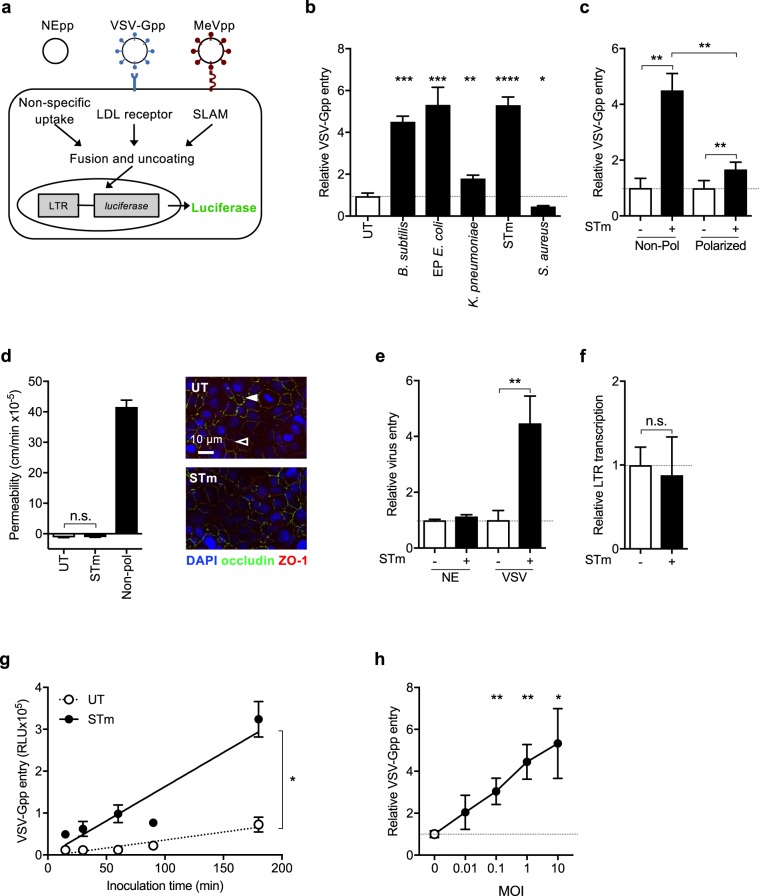
Diverse bacterial strains promote VSV-Gpp entry into epithelial cells. (**a**) Schematic diagram outlining lentiviral pseudoparticle (pp) infection. Particles lacking a viral envelope protein (NEpp) enter cells via non-specific phagocytic uptake mechanisms. In contrast, VSV glycoprotein (VSV-Gpp) or measles virus glycoprotein (MeVpp) expressing pp enter cells through specific receptors (LDL receptor or SLAMF1, respectively). Following glycoprotein-dependent fusion the lentivirus capsid uncoats, the genome replicates and expresses the reporter gene. (**b**) A549 lung epithelial cells were exposed to diverse bacterial strains: *B*. *subtilis;* enteropathogenic (EP) *E*. *coli; K*. *pneumoniae; STm* or *S*.*aureus* at a MOI of 10 for 1 h before inoculating with VSV-Gpp and antibiotic chloramphenicol (34 ug/mL; or tobramycin, 40 μg/mL, for chloramphenicol-resistant *P*. *aeruginosa*) and cultured for 48 h. Cells were lysed and luciferase activity measured and data expressed relative to untreated cells (UT). (**c**) Non-polarized (Non-pol) or polarized A549 cells were exposed to STm (MOI 10) for 1 h prior to inoculating with VSV-Gpp and chloramphenicol (34 μg/mL) for 48 h and infection quantified. (**d**) Polarized A549 cells were exposed to STm (MOI 10) for 1 h prior to apical addition of 70 kDa FITC-dextran and basolateral media sampled at various times to measure fluorescence and permeability, as a control non-polarized cells were evaluated. Polarized A549 cells were exposed to STm (MOI 10) for 1 h prior or left untreated (UT), fixed and stained for tight junction occludin (FITC – open arrow) and ZO-1 (PE- closed arrow) expression and imaged by confocal microscopy. (**e**) A549 cells were inoculated with STm (MOI 10) for 1 h before infecting with NE or VSV-Gpp particles for 48 h. (**f**) A549 cells were transfected with the lentiviral genome reporter DNA (pNL4.3env^−^rev^−^luc) for 8 h and treated with STm (MOI 10) and chloramphenicol for 16 h. (**g**) A549 cells were exposed to STm (MOI 10) for 1 h prior to inoculating with VSV-Gpp for defined periods of time and infection assessed after 48 h. (**h**) A549 cells were treated with STm at differing MOI, from 1 bacterium per 100 A549 cells to 10 bacteria per A549 cell, for 1 h prior to VSV-Gpp infection and culturing for 48 h in the presence of chloramphenicol. Bars represent mean ± S.D. for n = 3. Statistical comparison by unpaired t test where: n.s. p > 0.05; *p ≤ 0.05; **p ≤ 0.01; ***p ≤ 0.001 and ****p ≤ 0.0001. All data sets are representative of at least two independent experiments.

Lentiviruses can incorporate heterologous viral encoded glycoproteins into their envelope and the resulting pseudoparticles (pp) can be used to study the internalization pathway of diverse viruses (Fig. 1a). VSV is the prototypical Rhabdovirus that displays broad tissue tropism, defined by its encoded glycoprotein (G) and is widely used to target viral vectors for gene therapy purposes^17^. We screened Gram-positive and Gram-negative bacterial species for their effect on VSV-Gpp infection of A549 lung epithelial cells. *Bacillus subtilis* (Gram positive), *Escherichia coli* (Gram negative) and *Salmonella enterica* serovar Typhimurium (STm) (Gram negative) significantly increased VSV-Gpp infection whereas *Klebsiella pneumoniae* (Gram negative) and *Staphylococcus aureus* (Gram positive) had a minimal or negative effect (Fig. 1b). We selected STm for further studies to address the molecular mechanisms underlying this observation.

Polarized epithelia provide a barrier function to restrict macromolecular movement across mucosa and are well-recognized to limit virus infection^13,18^. A549 cells form tight junctions allowing us to investigate the effect of STm on VSV-Gpp infection in a polarized model system. STm promoted virus uptake in both non-polarized and polarized A549 (Fig. 1c), however, the effect was more pronounced in non-polarized cells. Polarized epithelial cells show a distinctive membranous staining of the tight junction proteins occludin and ZO-1. STm had no detectable effect on occludin or ZO-1 localization or A54 permeability (Fig. 1d), suggesting that changes in cellular permeability do not explain the observed pro-viral activity.

STm-mediated pp infection is dependent on viral glycoprotein-receptor interactions, as we failed to observe any increased uptake of glycoprotein-deficient lentivirus particles (NEpp) (Fig. 1e). Consistent with this interpretation STm had no effect on lentiviral promoter activity when the genome was transfected into cells and glycoprotein-receptor interactions bypassed (Fig. 1f). STm increased the rate of VSV-Gpp entry (Fig. 1g) and pseudoparticles expressing a GFP reporter showed that STm increased the frequency of VSV-Gpp infected cells (Supplementary Fig. S1). Furthermore, STm enhanced VSV entry at low multiplicities of infection (MOI) of 1 bacterium per 10 host cells (Fig. 1h).

**Figure 2:**
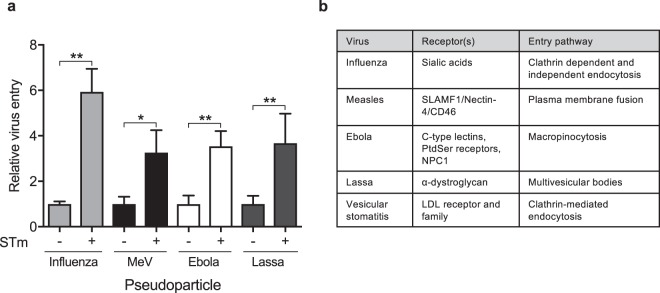
STm promotes virus entry into epithelial cells. (**a**) A549 cells were exposed to STm (MOI 10) for 1 h prior to inoculating with pseudoparticles expressing influenza (H7N1), measles (MeV, Edmonston), Lassa or Ebola glycoproteins and chloramphenicol (34 μg/mL) for 48 h and infectivity assessed. (**b**) To infect with MeV-pp A549 cells were engineered to express the receptor SLAMF1. Virus entry is expressed relative to untreated cells. Receptor dependency and internalization pathways of the viruses. Bars represent mean ± S.D. for n = 3. Statistical comparison by unpaired t test (*p ≤ 0.05 and **p ≤ 0.01). All data is representative of at least two independent experiments.

To determine whether the effect of STm was limited to VSV-Gpp, we screened pseudoparticles expressing influenza, Measles virus (MeV), Ebola or Lassa virus encoded glycoproteins that enter cells via distinct receptor-dependent pathways^12,19–21^. STm increased the entry of all the pseudoviruses tested (Fig. 2a), demonstrating a priming of diverse entry pathways that use clathrin-dependent and independent internalization routes (Fig. 2b). In summary, STm promotes the uptake of pseudoparticles expressing a range of viral glycoproteins that utilize diverse receptor-dependent pathways.

### Flagellin promotes viral entry

**Figure 3:**
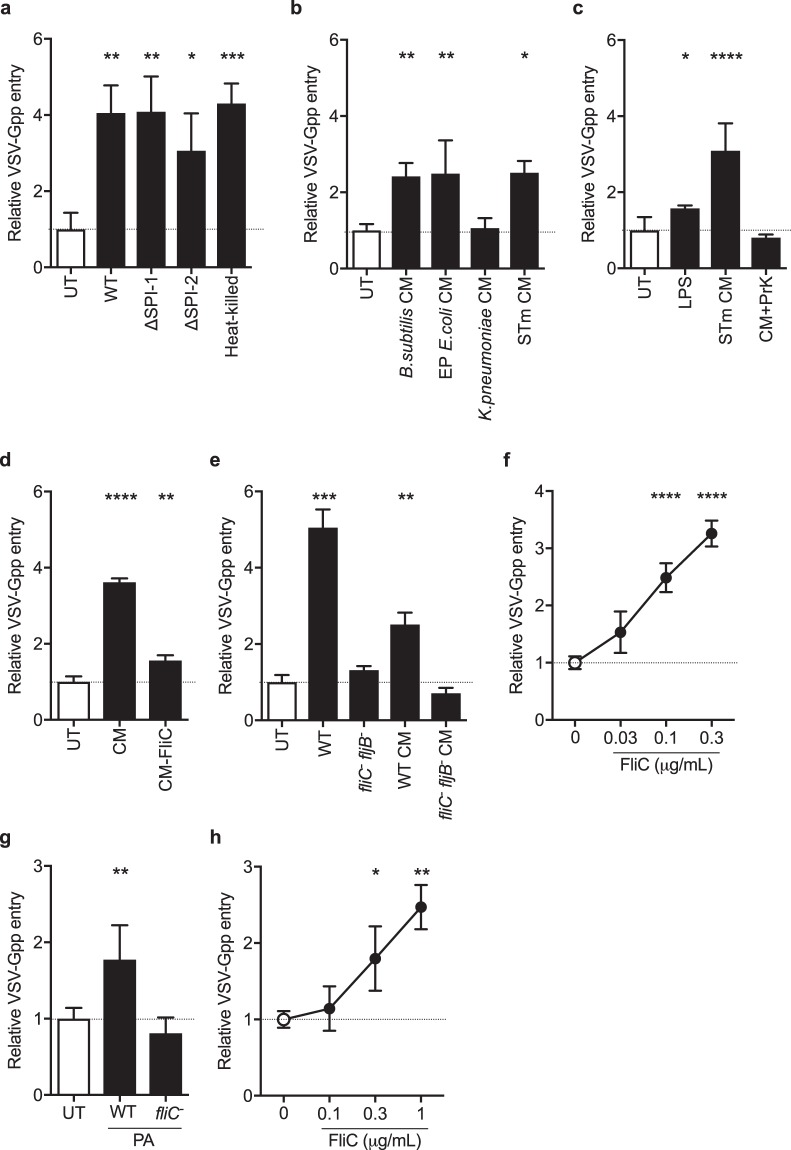
Bacterial flagellin promotes virus entry. (**a**) A549 cells were exposed to wild type (WT); SPI-1 deficient (∆SPI-1), SPI-2 deficient (∆SPI-2) or heat-killed (100 °C, 20 min) STm at an MOI of 10 for 1 h prior to inoculating with VSV-Gpp and chloramphenicol (34 μg/mL) and infection assessed after 48 h. (**b**) A549 cells were exposed to conditioned media (CM) from multiple strains: *B*.*subtilis*, enteropathogenic *E*.*coli*, *K*.*pneumoniae* or STm prior to inoculating with VSV-Gpp and entry determined. (**c**) A549 cells were exposed to STm LPS (10 μg/mL), untreated or proteinase K (PrK; 50 μg/mL) digested STm CM for 1 h prior to inoculating with VSV-Gpp and entry determined. (**d**) A549 cells were exposed to CM pre- and post-elution from an anti-FliC affinity chromatography column before inoculating with VSV-Gpp and entry determined. (**e**) A549 cells were exposed to WT or aflagellate (fliC-fljB-) STm (MOI 10) or CM from these strains for 1 h prior to inoculating with VSV-Gpp and tobramycin (40 μg/mL) and entry measured. (**f**) A549 cells were exposed to STm flagellin (FliC) at various concentrations for 1 h and inoculated with VSV-Gpp and entry assessed. (**g**) A549 cells were exposed to WT or aflagellate (fliC-) *P*.*aeruginosa* (PA) (MOI 10) (**h**) or *P*.*aeruginosa* FliC at various concentrations for 1 h prior to inoculating with VSV-Gpp and tobramycin (40 μg/mL) and entry determined. Bars represent mean ± S.D. for n = 3. Statistical comparison by unpaired t test where: n.s. p > 0.05; *p ≤ 0.05; **p ≤ 0.01; ***p ≤ 0.001 and ****p ≤ 0.0001. All data sets are representative of at least two independent experiments.

The infectivity and pathogenicity of STm is influenced by *Salmonella* pathogenicity islands (SPI), particularly SPI-1 and SPI-2^22^. STm lacking these two pathogenicity islands increased VSV-Gpp entry to a comparable extent as wild type (Fig. 3a), suggesting that the capacity of STm to boost virus internalization is not dependent on its ability to invade epithelial cells. Furthermore, heat-killed STm promoted VSV-Gpp entry at comparable levels to live bacteria, implying that *de novo* protein expression by bacteria after exposure to epithelial cells was not necessary to promote viral uptake (Fig. 3a). This finding, in conjunction with the modest numbers of bacteria (MOI < 0.1, Fig. 1h) required to promote viral entry suggested that a soluble factor(s) in the extracellular milieu may be responsible for the effect. We therefore examined whether conditioned medium (CM) in which bacteria had been grown could promote virus entry. CM from *B*. *subtilis*, *E*. *coli* and STm cultures, but not from *K*. *pneumoniae*, promoted VSV-Gpp infection (Fig. 3b), suggesting the presence of a soluble factor(s).

A potential candidate molecule is LPS, however, treating epithelial cells with LPS had a modest effect on VSV-Gpp uptake (Fig. 3c). Proteinase K digestion of STm CM abrogated its activity, demonstrating a proteinaceous factor (Fig. 3c). Mass spectrometric analysis of STm CM identified flagellin as one of the most abundantly expressed proteins (Table S1) and immunodepletion of flagellin^23^ using a specific monoclonal antibody (CM-FliC) reduced the pro-viral activity of CM (Fig. 3d). Furthermore, STm lacking both flagellin genes (*fliC* and *fljB*), or CM in which it had been grown, had no effect on VSV-Gpp uptake (Fig. 3e). Treating A549 lung epithelial cells with recombinant flagellin (FliC) induced a dose-dependent increase in VSV-Gpp entry (Fig. 3f). The role of flagellin in this process was confirmed using the flagellated, Gram-negative, opportunistic pathogen *Pseudomonas aeruginosa* (Fig. 3g). The flagellated parent strain promoted viral entry, whilst an aflagellate mutant lost this ability, furthermore stimulating A549 cells with purified flagellin from *P*.*aeruginosa* increased viral entry in a dose-dependent manner (Fig. 3h). In summary, flagellated Gram-positive and Gram-negative bacteria, or purified flagellin alone, promoted viral entry into epithelial cells.

### Flagellin promotes temperature-dependent virus cell association and internalization

**Figure 4:**
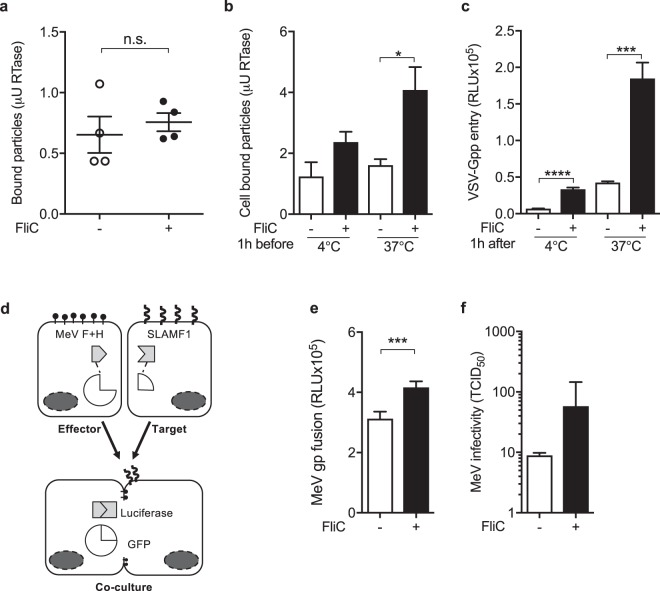
Flagellin augments pseudoparticle infection post-attachment. (**a**) To assess whether flagellin can directly bind VSV-Gpp the virus was incubated with histidine tagged flagellin (0.3 μg/mL) for 1 h, complexes captured with Ni-NTA agarose beads and viral associated RTase activity measured. (**b**) To determine whether flagellin augments virus attachment A549 cells were treated with flagellin (0.3 μg/mL) at 37 °C for 1 h and inoculated with VSV-Gpp for 4 °C or 37 °C for 1 h, unbound virus removed by extensive washing and cell-associated viral RTase activity measured. (**c**) To ascertain whether flagellin can promote infection post virus-cell attachment, A549 cells were incubated with VSV-Gpp for 1 h at 4 °C or 37 °C, unbound virus removed by washing and cells treated with flagellin (0.3 μg/mL) and cultured for 48 h before measuring infectivity. (**d**) Schematic diagram outlining the principles of the MeV cell-cell fusion assay. To determine fusion, MeV F and H glycoproteins are expressed in effector cells along with an enzymatically inactive split GFP-renilla luciferase. In parallel, the receptor SLAMF1 is delivered into target cells with the remaining half of the GFP-renilla reporter. After co-culturing effector and target cells, F and H receptor engagement triggers cell-cell fusion, GFP-Renilla reconstitution and reporter gene activity. (**e**) Target cells were treated with STm FliC (1 μg/mL) for 1 h and co-cultured with effector cells for 24 h and lysed to read luciferase activity. (**f**) A549-SLAM cells were treated with flagellin (1 μg/mL) for 1 h and infected with Measles virus (MOI 0.1) for 24 h, lysed and the infectivity of cell-associated virus determined (tissue culture infectious dose - TCID_50_/mL). Bars represent mean ± S.D. for n = 3. Statistical comparison by unpaired t test where: *p ≤ 0.05; **p ≤ 0.01; ***p ≤ 0.001 and ****p ≤ 0.0001. All data sets are representative of at least two independent experiments.

To ascertain whether flagellin can interact directly with lentiviral pseudoparticles, VSV-Gpp was incubated with histidine tagged FliC (His-FliC) and complexes captured with nickel-agarose beads. Lentiviruses were detected using a PCR assay that quantifies particle-associated reverse transcriptase^24^. We previously reported that nickel-agarose beads could efficiently capture His-Flic^25^ and yet FliC did not promote VSV-Gpp binding to the nickel-sepharose beads (Fig. 4a), suggesting that flagellin does not bind virus glycoprotein expressing lentivirus particles in any appreciable manner.

Many viruses initiate infection by low-affinity interactions with cell surface expressed heparin-sulphate proteoglycans and we investigated the effect of flagellin on virus-cell attachment. Virus internalization is temperature dependent and incubating virus-inoculated cells at 4 °C leads to an accumulation of particles at the cell surface. Flagellin had no effect on the levels of cell bound VSV-Gpp at 4 °C; however, at the entry-permissive temperature of 37 °C we noted a 2-fold increase in cell-associated VSV-Gpp particles (Fig. 4b), supporting a role for flagellin in promoting VSV-Gpp internalization. To investigate further we assessed the activity of flagellin to augment virus infection after viral adsorption to the target cells. Flagellin increased VSV-Gpp infection post-viral attachment (Fig. 4c) with comparable activity following virus adsorption at 4 °C or 37 °C, demonstrating a role for flagellin to potentiate viral internalization. VSV enters cells via clathrin-mediated endocytosis and is dependent on a dynamic actin network^12^. We confirmed that chloroquine, a lysosomotropic agent that inhibits endosomal acidification, reduced VSV-Gpp infection in the presence or absence of flagellin (Supplementary Fig. S2a). Furthermore, flagellin had no effect on VSV-Gpp sensitivity to inhibition (cytochalasin D) or promotion (jasplakinolide) of the actin polymerization (Supplementary Fig. S2a), suggesting that flagellin has a minimal effect on VSV-Gpp cellular uptake pathway(s). Importantly, the pharmacological inhibitors had no detectable effect on flagellin induced interleukin 8 (IL8) expression (Supplementary Fig. S2b). We were interested in assessing the impact of flagellin on transferrin uptake, a classical ligand for clathrin-dependent endocytosis. We observed a temperature and time-dependent internalization of transferrin into A549 cells that was not affected by prior treatment with flagellin (Supplementary Fig. S3), suggesting that the complex multivalent virus-receptor interactions are not recapitulated with a monomeric ligand such as transferrin.

Many viruses disseminate and infect new target cells by inducing the fusion of neighbouring cells in the absence of virus particle endocytosis, e.g. MeV. To investigate whether flagellin could modulate MeV induced cell-cell fusion we developed a quantitative reporter assay (Fig. 4d) and demonstrated a significant increase in SLAM-dependent fusion when target cells were treated with flagellin (Fig. 4e). To determine whether flagellin-induced MeV fusion or pp uptake (Fig. 2a) associates with increased viral replication and production of nascent progeny we used virulent MeV infection of A549-SLAMF1 cells as a model. Flagellin increased the level of infectious virions produced from infected cells (Fig. 4f). This observation demonstrates that flagellin signalling is effective at the plasma membrane and does not require vesicular trafficking or endocytosis.

### Flagellin increases virus entry through NF-ΚB and TLR5

**Figure 5:**
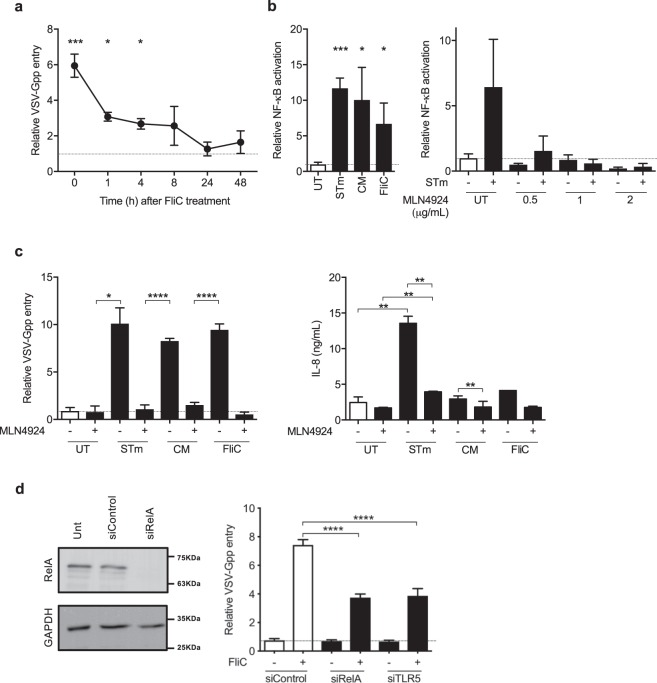
Flagellin increases virus entry through NF-kB and signalling. (**a**) A549 cells were treated with STm FliC (1 μg/mL) for 1 h and media replaced for various time periods prior to inoculating with VSV-Gpp for 1 h and luciferase expression assessed after 48 h. (**b**) A549 cells were transfected with an NF-ΚB reporter plasmid (pConA-luciferase) for 24 h, inoculated with STm (MOI 10), STm CM or STm FliC (0.3 μg/mL) for 1 h prior to chloramphenicol (34 μg/mL) addition and NF-ΚB activity measured after 24 h. NF-ΚB reporter transfected cells were treated with MLN4924 for 15 mins and inoculated with STm (MOI 10) for 1 h prior to chloramphenicol (34 μg/mL) addition and NF-ΚB activity measured. (**c**) A549 cells were treated with MLN4924 (1 uM) for 15 mins and inoculated with STm (MOI 10), STm CM or STm FliC (0.3 μg/mL) for 1 h prior to inoculating with VSV-Gpp and chloramphenicol (34 μg/mL) and VSV-Gpp entry or interleukin 8 (IL-8) expression. (**d**) A549 cells were transfected with control siRNA or RelA siRNA for 24 h prior to lysing for western blotting. Transfected cells were exposed to STm flagellin (0.3 μg/mL) for 1 h prior to inoculating with VSV-Gpp and entry determined. Bars represent mean ± S.D. for n = 3. Statistical comparison by unpaired t test where: n.s. p > 0.05; *p ≤ 0.05; **p ≤ 0.01; ***p ≤ 0.001 and ****p ≤ 0.0001. All data sets are representative of at least two independent experiments.

Flagellin increased viral entry for at least 4 h following the initial exposure of the epithelial cells to the protein, suggesting that it induces a lasting signalling effect (Fig. 5a). Flagellin is sensed by two pathways; TLR5 and the NAIP/Nlrc4-inflammasome, and both pathways activate NF-ΚB^26^. We confirm that STm, CM and purified flagellin activated an NF-ΚB reporter in A549 cells (Fig. 5b). MLN-4924 inhibits the Nedd8-activating enzyme that regulates Cullin-RING ubiquitin ligases^27^ and has been reported to limit NF-ΚB signalling pathways^28,29^ therefore we evaluated its effect on flagellin induced NF-ΚB signaling. We confirmed that MLN4924 inhibits STm-dependent NF-ΚB activation^16^ (Fig. 5b). Importantly, treating A549 cells with MLN4924 ablated both the pro-viral and pro-inflammatory activities of STm, CM and flagellin (Fig. 5c). Of note, MLN-4924 had no effect on VSV-G infection in the absence of STm, suggesting a minimal role for NF-ΚB signaling in the basal entry process of this virus (Fig. 5c). To confirm a role for NF-ΚB signaling and TLR5 activation in flagellin-boosted viral entry, we used siRNA to knock down RelA or TLR5 in A549 cells. We observed a significant reduction in RelA expression following silencing, however, given the lack of antibodies specific for TLR5 we were only able to quantify mRNA species by PCR and noted a 30% reduction. Both siRNA treatments reduced the ability of flagellin to boost VSV-Gpp (Fig. 5d), suggesting a role for NF-ΚB and TLR5 in this pathway.

## Discussion

In this study we examined the interplay between different bacterial species and the uptake pathway of pseudovirus particles^12,19–21^. Our results provide the first mechanistic evidence for flagellin to promote the uptake of particles expressing a range of viral glycoproteins that engage different cellular receptors into A549 epithelial cells. The evidence that flagellin was the bacterial component responsible is unequivocal and includes: i) loss of flagellin from *P*. *aeruginosa* and STm abrogates the phenotype; ii) purified flagellin from these species promotes viral entry. We noted differences in the ability of different bacterial species to promote virus entry and this is likely to reflect the pattern and frequency of flagellin expression and flagella formation. For instance, *B*. *subtilis*, *E*. *coli* and STm can express multiple flagella in a peritrichous distribution^30^, whereas *P*. *aeruginosa* expresses a single, polar flagellum, most likely explaining the differences in pro-viral activity observed between *P*. *aeruginosa* and STm. Importantly, purified flagellin from both *P*. *aeruginosa* and STm showed comparable ability to increase VSV-Gpp uptake in a concentration-dependent manner. Since the pro-viral effects were seen using heat-killed bacteria or purified bacterial flagellin, antibiotic use is unlikely to influence the pro-viral effects of bacterial species. The pro-viral activity of flagellated STm is long-lasting and detectable at low MOI, suggesting that flagellin induces a bystander effect and, even in sites sparsely colonized by flagellated bacteria, may regulate epithelial cell permissivity. We recognize the limitations of our study that utilized pseudovirus particles to investigate entry pathway of a range of viruses, highlighting the need for future research with complete replication systems to assess the effect(s) of flagellin on the full viral life cycle.

Host receptor mobility in the plasma membrane has been reported to regulate virus internalization rates^31,32^. We noted a temperature-dependent effect of flagellin to augment VSV-Gpp cell-association that may reflect a requirement for active receptor diffusion at the plasma membrane. Of note, we previously reported a role for tumor necrosis factor and related family members to induced NF-ΚB signaling to regulate the lateral diffusion of host tetraspanin CD81 via cytoplasmic tail association with the actin cytoskeleton^33,34^, providing a mechanism for flagellin to regulate membrane protein dynamics. The ability of flagellin to promote MeV encoded glycoprotein-SLAMF1 cell-cell plasma membrane fusion lends further support to a model where flagellin signalling is effective at the plasma membrane and does not require vesicular trafficking or endocytosis.

Gene silencing and the use of the inhibitor MLN4924 support a role for TLR5 and NF-ΚB signaling in promoting viral uptake. What mechanistic role these molecules play in the viral entry process remains to be determined in future studies, especially as LPS can activate NF-ΚB signaling and yet this TLR ligand had a negligible effect on viral entry. Engagement of this cascade can induce a range of cellular changes. Whilst TLR5 ligation can promote the expression of IL-8 and other cytokines it can have other consequences. Possibly relevant to the current study is the observation that TLR5 enhanced antigen capture and presentation through MHC-II occurs in a MyD88 independent manner^35^. The A549 lung epithelial cells used in this study express both MHCI and MHCII^36^. Thus, it is possible that flagellin ligation of TLR5 promotes immunological changes that indirectly regulate virus internalization.

In order for TLR5 to be relevant for viral uptake one would hypothesize it should be expressed at the apical surface of epithelial cells. TLR5 was initially reported as being restricted to the basolateral side of epithelial cells^37^. However, subsequent studies report that TLR5 is expressed throughout the surface of epithelial cells. For instance, in the lung epithelium, TLR5 is expressed at apical and basal surfaces^38^. Indeed, it is likely that ligation of TLR5 on the lung epithelia is essential to drive the early recruitment of immune cells into the airways following flagellin administration^39^. Thus, in sites like the lung, exposure of TLR5 to flagellin is unlikely to require local damage of the epithelial layer to promote viral uptake.

Flagellin has been shown to have remarkable effects on a range of cell types, and changes in TLR5 are associated with both increased and decreased risk of many chronic diseases, including infection, colitis and cancer^38^. The effects of TLR5 ligation can be rapid and result in changes in cell behavior, mobility and cytokine production^40,41^. Moreover, loss of TLR5 in mice is associated with reduced immune responses to flu vaccine^42^, suggesting that the levels of flagellin expressed by the microbiome are sufficient to influence the host immune response. This is important because most reports, including many by us, study the response to purified flagellin protein and often at supraphysiological concentrations. *In vivo* mouse studies show that immunization with flagellin limits influenza or rotavirus infection^43,44^, most likely mediated via the increased recruitment and cytokine activity of immune cells in the local environment. As such, purified flagellin has great potential as an adjuvant and carrier protein for use in vaccination regimens to protect against influenza^45^. However, this may differ to how flagellin acts when expressed by bacteria in the gut or other niches and it is often difficult to discriminate the effects of flagellin on individual cell types in such *in vivo* studies. Hence in this study we took a more reductionist approach to identify the effect of flagellin on virus infected epithelial cells. It is interesting to note that flagellin was reported to increase human immunodeficiency virus (HIV-1) replication in lymphoid tissue *ex vivo*^46^ and to reactivate latent HIV provirus from T cells^47^. We have confirmed and extended these results to show a role for flagellin to augment HIV entry into CD4^+^ T cells (unpublished observations). Our observations highlight the need for further studies to assess the impact of flagellin on viral tropism and replicative burden within the local environment.

Many bacterial species that are pathogens or normal components of the gut microbiome are flagellated and express flagellin that can ligate TLR5, including *Salmonella enterica*, *E*. *coli*, *Clostridium difficile* and *Listeria monocytogenes*. It is noteworthy that no common bacterial pathogen of the healthy lung is flagellated. *P*. *aeruginosa*, though flagellated, is a pathogen of individuals who are typically immunocompromised, such as patients with bronchiectasis. In such individuals, the burden of *P*. *aeruginosa* in the lung can often reach >10^8^ bacteria/mL of sputum^48^, providing a reservoir of flagellin production that may contribute to the increased burden of viral infections seen during bronchiectasis^49,50^. Other, atypical flagellated respiratory pathogens that affect immunocompromised individuals include pathogens that cause Legionnaire’s disease and melioidosis and it is noteworthy that TLR5 was reported as a risk factor for defining outcome in these infections^51,52^. Collectively these studies show that flagellin can fine-tune the local susceptibility to infection and immunity in sites such as the lung. This may be most prominent in the aged where a relative decrease in the functionality of TLRs, with the exception of TLR5, has been reported^53^. Thus, if TLR5 activity is maintained flagellin may enhances susceptibility to viral infection in individuals with a diminished anti-viral-associated TLR activity related to immune senescence. In summary, this study provides a mechanism by which the risk of viral infection may be altered by changes in the microbial community that associates with the mucosa, or through the presence of flagellated pathogens infecting these sites.

## Materials and Methods

### Cell culture, reagents and pharmacological inhibitors

A549 human lung epithelial carcinoma cells and 293 T human embryonic kidney cells (ATCC) were maintained in DMEM supplemented with 10% FBS/1% non-essential amino acids/1% L-glutamine and 50 units/mL penicillin/streptomycin. A549 cells expressing the MeV virus receptor signalling lymphocytic activation molecule F1 (SLAMF1) were produced by HIV-1 based transduction using a puromycin resistant bi-cistronic transcriptional cassette and maintained in 10% FBS/DMEM supplemented with 1 μg/mL puromycin (Gibco). A549 cells were allowed to polarize and permeability assessed by monitoring dextran flux. STm flagellin (FliC) was purified as previously described^25^ and commercial supplies of LPS (Enzo Life Sciences). *P*. aeruginosa flagellin (PA-Fla) (Invivogen) and MLN-4924 (Calbiochem) used. All pharmacological inhibitors Chloroquine, Cytochalasin D and Jasplakinolide (Calbiochem) were tested for cytotoxicity prior to use using a 3-(4,5-Dimethylthiazol-2-yl)-2,5-Diphenyltetrazolium Bromide (MTT) assay to assess cell viability.

### Epithelial permeability

Paracellular permeability was quantified by measuring the transepithelial flux of a 70 kDa FITC-labelled dextran (Sigma Aldrich) as previously described^18^. Briefly, cells were polarized on 0.4 μm pore-size Transwell PET membranes and were apically inoculated with STm (MOI 10) before the application of FITC-dextran. Samples were removed from the basolateral chamber over 30 min and fluorescence measured to determine rate of permeability.

### Confocal imaging of tight junction proteins

Cells were grown on Nunc Thermanox coverslips (Thermo Fisher Scientific), inoculated with STm (MOI 10) for 1 h and fixed with 4% paraformaldehyde. Tight junction proteins were visualized by staining with a primary mouse α-occludin or rabbit α-ZO-1 (2 μg/mL; Invitrogen) followed by secondary goat α-mouse Alexa Fluor 488 or goat α-rabbit Alexa Fluor 594 (Invitrogen). Nuclei were counterstained with DAPI and cells imaged using a Zeiss 510 Meta confocal microscope.

### Bacterial strains and culture

STm wild type strain SL1344, aflagellate strain SL1344 (*fliC*::*cat fljB*::*aph)*, SPI-1 mutant SL1344 SPI-1::*aph* and SPI-2 mutant SL1344 *ssaV*::*aph* were used together with *B*. *subtilis* NCTC 3610, EP *E*. *coli* O127:H6 E2348/69, *K*. *pneumoniae* NCTC 9633, *P*. *aeruginosa* wild type strain PA14 and aflagellate strain PA14 *fliC*::*Tn* Gm15^54^ and *S*. *aureus* NCTC 8532. Bacteria were cultured in Luria Bertani (LB; Sigma-Aldrich) broth at 37 °C with aeration, harvested at mid-log phase (OD_600_ 0.5), washed three times in phosphate buffered saline and resuspended in 10% FBS/DMEM prior to inoculating epithelial cells. Bacteria were heat killed at 100 °C for 20 min and the absence of viable cells confirmed by overnight culture on LB plates.

### Generation of bacterial conditioned media

Bacteria were cultured in DMEM overnight, the media clarified by low speed centrifugation and passed through 0.22 μm filters. The absence of viable cells was confirmed by overnight culture on LB plates. Conditioned media (CM) was treated with Proteinase K (50 μg/mL; Sigma Aldrich) for 30 min and the enzyme heat inactivated at 100 °C for 5 min. Flagellin was depleted from the CM by passing over an affinity column with an α-FliC monoclonal antibody^25^.

### Mass spectrometry

To prepare CM for mass spectrometry, 50 mL STm CM using phenol red free DMEM (Gibco) was centrifuged in a 100 kDa vivaspin concentration column (Sartorius Stedim Biotech), the top fraction was dialysed in a 10 kDa Slide-A-Lyzer cassette (Thermo Fisher Scientific) in ddH_2_O and tested for pro-viral activity. Samples were sent to the Advanced Mass Spectrometry Facility, UoB, to assess protein fragments present using an Orbitrap mass spectrometer and the Sequest algorithm used to identify STm encoded proteins.

### Pseudoparticle infection

Pseudoparticles were generated as previously described^55^ using plasmids encoding a HIV provirus expressing luciferase and viral envelope glycoproteins from Ebola (Zaire Mayinga strain), influenza H7 and N1, Lassa, MeV (Edmonston strain) F/H and VSV (Indiana strain) G or no envelope (NE) control. A549 cells were inoculated with bacteria or treated with CM, LPS or flagellin for 1 h. Pseudoparticles were added with 34 μg/mL chloramphenicol (Sigma Aldrich) or 40 μg/mL tobramycin (Sigma Aldrich) and the cells incubated for 48 h prior to lysis and measurement of luciferase activity. Viral-glycoprotein specific particle entry was calculated by subtracting the no-envelope signal and expressing relative to untreated control values. To determine the rate of virus entry, A549 cells were inoculated with STm (MOI 10) for 1 h prior to VSV-Gpp addition, washed three times with media to remove unbound virus at time points specified and incubated with fresh media for 24 h. To assess the effect of flagellin on virus internalization post-attachment, cells were incubated with pseudoparticles at 4 °C or 37 °C for 1 h, unbound virus removed by washing and cells treated with flagellin at 37 °C for 48 h to determine virus infectivity.

### Flagellin-pseudoparticle binding assay

Purified histidine-flagellin (1 μg/mL) was incubated with VSV-Gpp for 1 h at 37 °C, then mixed with Ni-NTA agarose beads for 1 h at room temperature and loaded into a polypropylene column. The beads were washed 5x with PBS prior to eluting the flagellin with imidazole (100 mM) and samples collected from all elutions. The flow through, washes and elutions were assessed for pseudoparticles by product enhanced reverse transcriptase (PERT) assay.

### PERT assay

Lentivirus associated reverse transcriptase (RTase) was detected as previously described^24^. Extracellular virus or whole cells were lysed with an equal volume of 2x lysis buffer (0.25% triton X-100, 50 mM KCl, 100 mM TrisHCl pH 7.4, 40% glycerol) and added to the reaction mix (QuantiTect kit, Qiagen) containing forward and reverse MS2 primers, MS2 RNA and RNase inhibitor. A standard curve was prepared using HIV-1 RTase to determine units (U) of RTase activity. The thermal cycler conditions were 10 min at 45 °C, 15 min at 95 °C and 50 cycles of amplification, consisting of 10 s at 95 °C and 30 s at 60 °C. Pseudoparticle attachment to cells was measured by lysing the cells to quantify particle associated RTase.

### MeV cell-cell fusion reporter assay

Cell-cell fusion induced by the expression of MeV F and H proteins was quantified as previously described^56^. Briefly, target cells were transfected with one half of a split dual reporter, encoding eGFP and *Renilla reniformis* luciferase^57^, and the MeV receptor SLAMF1, while effector cells were transfected with the complimentary part of the dual reporter and viral glycoproteins. Target cells were treated with flagellin (1 μg/mL) for 1 h prior to culturing with effector cells for 24 h and luciferase activity quantified as a measure of fusion.

### MeV infection

A549-SLAMF1 cells were pre-treated with flagellin (1 μg/mL) and infected with a recombinant MeV strain IC323 expressing eGFP^58^ at a MOI of 0.01. Five days post-infection, cells were frozen at −80 °C and thawed at room temperature and the supernatants spin clarified (3000 × g, 30 min) and their infectious titer for Vero-SLAMF1 cells measured.

### NF-ΚB reporter assay

A549 cells were transfected according to manufacturer’s instructions (Fugene 6; Promega) with plasmid pConA-luciferase and incubated for 24 h prior to treatment with flagellin.

### RelA and TLR5 silencing

A549 cells were transfected according to manufacturer’s instructions (Dharmafect 1; Thermo Scientific) with ON-TARGET plus smart pool siRNA targeting RelA, human TLR5 or a non-targeting control 24 h prior to treating with flagellin and pp infection. Silencing was assessed by PCR/western blotting. RNA was prepared using the Qiagen RNeasy kit and amplified for TLR5 using human TLR5 primer pair Hs01019558_m1 (Thermo Scientific) in a quantitative reverse-transcription PCR (qRT-PCR) in accordance with the manufacturer’s guidelines (CellsDirect kit; Thermo Scientific) and fluorescence monitored in a 7500 real-time PCR machine (Applied Biosystems). Glyceraldehyde 3-phosphate dehydrogenase (GAPDH) was included as an endogenous control for amplification efficiency, and TLR5 amplification normalized to GAPDH using the ΔΔCt method. Proteins were separated using an 8% SDS-PAGE gel, transferred to PVDF membrane (Millipore) and probed using a primary RelA (p65) rabbit monoclonal antibody (Cell Signaling Technology) or α-actin mouse monoclonal antibody (Sigma Aldrich) and secondary donkey α-rabbit HRP antibody (GE Healthcare) or sheep α-mouse HRP (GE Healthcare). Membranes were incubated in EZ-ECL prior to image capture using a PXi Multi-application gel imaging system (Syngene).

### Statistical analysis

Statistical analyses were performed using Student’s *t* test in Prism 6.0 (GraphPad), with *P* < 0.05 being considered statistically significant and corrected for multiple comparisons when required (Bonferroni). Error bars show standard deviation and data are representative of two or more independent replicates.

## Supplementary information

Supplementary Information

## Data Availability

The data sets generated during the current study are available from the corresponding author on reasonable request.
