# Supplementary material for: Bacterial flagellin promotes viral entry via an NF-kB and Toll Like Receptor 5 dependent pathway
Source: Sci Rep. 2019 May 27;9:7903. doi: 10.1038/s41598-019-44263-7 (PMC6536546; doi:10.1038/s41598-019-44263-7)
Supplement: Supplementary file 1 — Supplementary Information [file 41598_2019_44263_MOESM1_ESM.pdf]

Supplementary Information

Bacterial flagellin promotes viral entry via an NF-kB and Toll Like Receptor 5 dependent pathway.

Elizabeth K Benedikz[1,2], Dalan Bailey[1,3], Charlotte NL Cook[2],
Daniel Gonçalves-Carneiro[1], Michelle MC Buckner[2], Jessica MA Blair[2],
Timothy J Wells[2], Nicola F Fletcher[1], Margaret Goodall[1], Adriana Flores-Langarica[1],
Robert A Kingsley[4], Jens Madsen[5], Jessica Teeling[6], Sebastian L Johnston[7],
Calman A MacLennan[8], Peter Balfe[1], Ian R Henderson[2], Laura J V Piddock[2],
Adam F Cunningham[1,2] and Jane A McKeating[1,9] *

1. Institute of Immunology and Immunotherapy, University of Birmingham, UK.
2. Institute of Microbiology and Infection, University of Birmingham, UK.
3. The Pirbright Institute, Guildford, Surrey, UK.
4. Institute of Food Research, Norwich, UK.
5. Department of Child Health, University of Southampton, Southampton, UK.
6. Biological Sciences, University Hospital Southampton NHS Foundation Trust, Southampton, UK.
7. National Heart and Lung Institute, Imperial College London, London, UK.
8. Jenner Institute, Nuffield Department of Medicine, University of Oxford, UK
9. Nuffield Department of Medicine, University of Oxford, UK.

* Corresponding author

Contents :

Fig.S1. Flagellin increases the frequency of VSV-Gpp infected cells.

Fig.S2. Flagellin has a minimal impact on VSV sensitivity to pharmacological inhibition of cellular uptake pathways.

Fig.S3. Flagellin has a minimal impact on Transferrin uptake.

Fig.S4. Uncropped original images used in figure 5a

Table S1. The top 10 protein fragments found by semi-quantitative mass spectrometry of STm CM.

Fig.S1

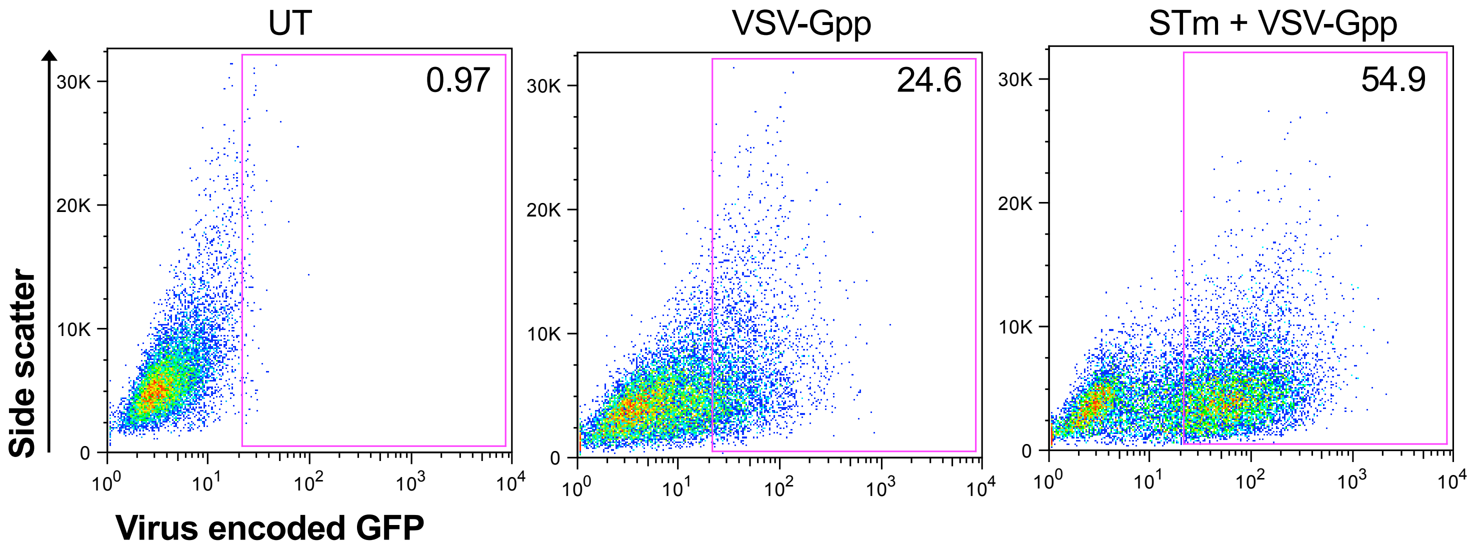

Flagellin increases the frequency of VSV-Gpp infected cells. A549 cells were exposed to STm (MOI 10) for 1h prior to inoculating with VSV-Gpp containing a GFP reporter and chloramphenicol and cultured for 48h. Viral glycoprotein-mediated entry was assessed by flow cytometry. Gate shows frequency of GFP^+ cells in live cell population.

Fig.S2

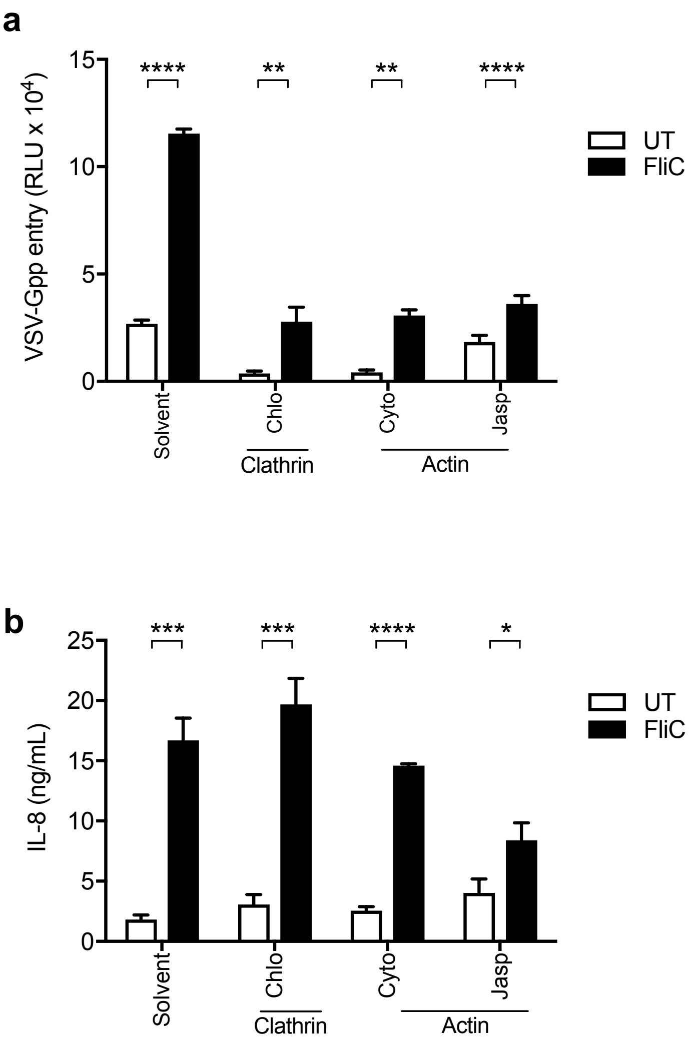

Flagellin has a minimal impact on VSV sensitivity to pharmacological inhibition of cellular uptake pathways. A549 cells were treated with chloroquine (Chlo: 50 µg/ml), Cytochalasin D (Cyto: 10 µg/ml), Jasplakinolide (Jasp: 10 µg/ml) or solvent alone for 30 minutes prior to treating with FliC (0.3 µg/ml) for 1h and inoculating with VSV-Gpp for 1h. After 48h the cells were lysed to quantify luciferase-dependent viral entry (**a**) and secreted IL8 (**b**). Error bars show SD of 3 technical replicates, statistical comparison by unpaired t test: * $P \leq 0.05$; ** $P \leq 0.01$; *** $P \leq 0.001$ and **** $P \leq 0.0001$.

Fig.S3

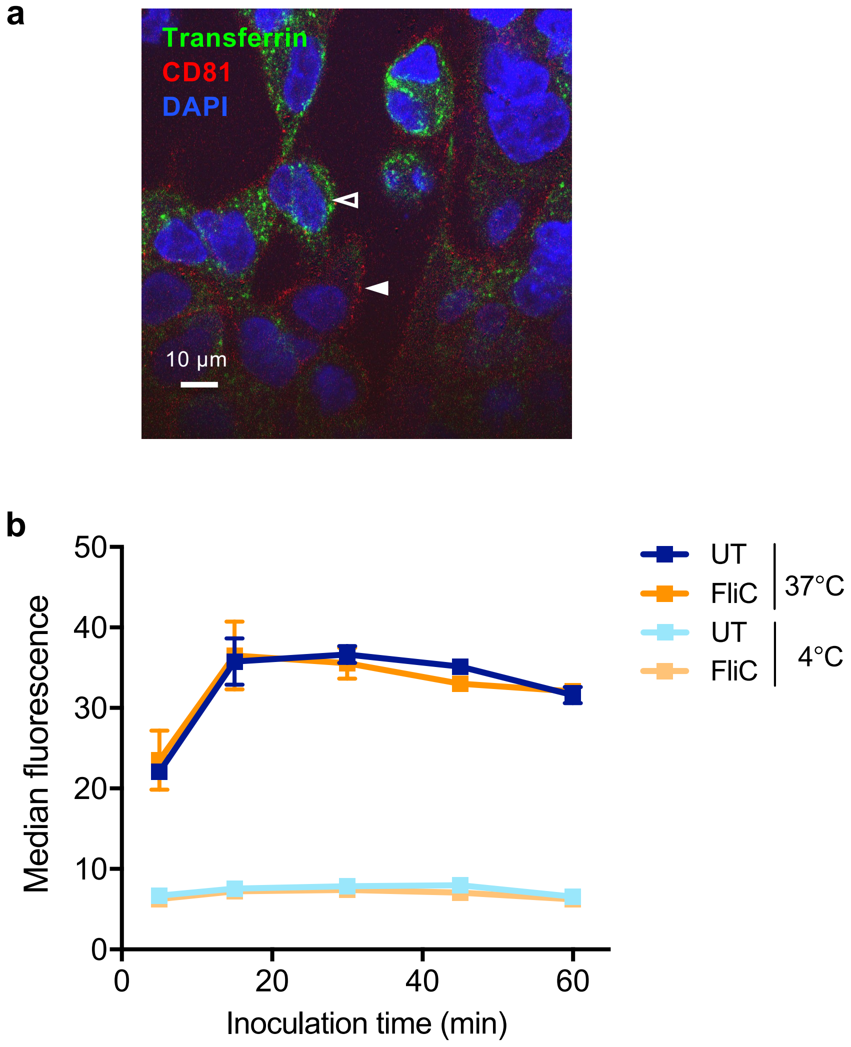

Flagellin has a minimal impact on Transferrin uptake. A549 cells were incubated with Alexa Fluor 488 conjugated Transferrin (Tf at 10 µg/mL) (open arrow head) for 1h, fixed and stained for plasma membrane CD81 (closed arrow head) expression, nuclei stained with DAPI and cells imaged by confocal microscopy (a). A549 cells were treated with FliC (1 µg/mL) for 1h at 37°C prior to incubating with Tf at 37 °C or 4 °C at the indicated time points. Cells were fixed and analysed by flow cytometry. Representative of 3 biological replicates, error bars show SD of 2 technical replicates.

Fig.S4

a

RelA WB

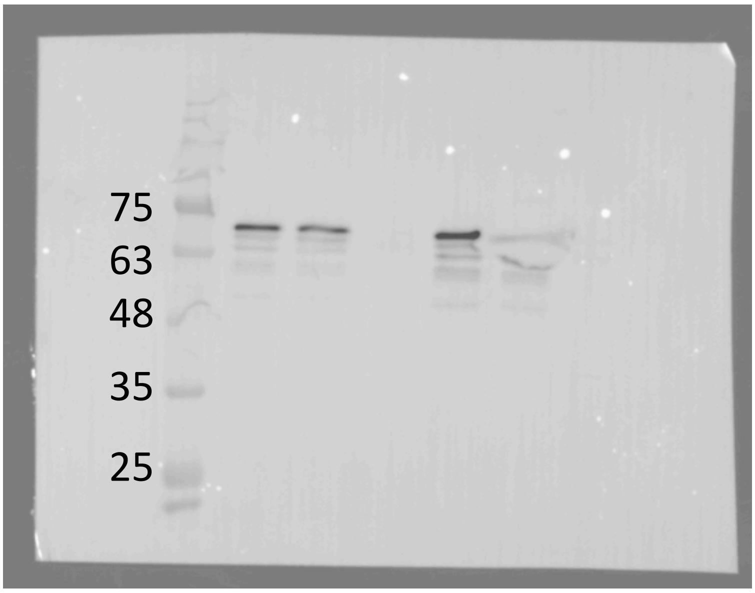

b

GAPDH WB

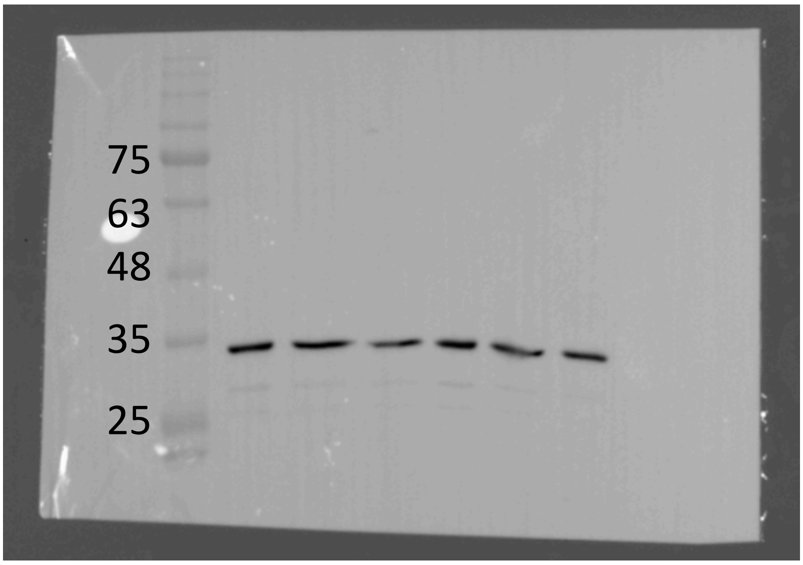

Uncropped original images used in figure 5a

Table S1. The top 10 protein fragments found by semi-quantitative mass spectrometry of STm CM.

	Accession	Description	Score
1	Q9R4Q7	Flagellin OS=Salmonella typhimurium PE=4 SV=1 - [Q9R4Q7_SALTM]	285.1873
2	A0FLJ5	Phase I flagellin middle domain variant C174 OS=Salmonella typhimurium GN=fliC PE=4 SV=1 - [A0FLJ5_SALTM]	257.2394
3	T5K278	Flagellin (Fragment) OS=Salmonella enterica subsp. enterica serovar Typhimurium str. STm3 GN=B573_24790 PE=4 SV=1 - [T5K278_SALTM]	193.9643
4	A0A038EDW8	Flagellin (Fragment) OS=Salmonella enterica subsp. enterica serovar Typhimurium str. 108402 GN=N936_02335 PE=4 SV=1 - [A0A038EDW8_SALTM]	166.02
5	A0A023SY02	Membrane protein OS=Salmonella typhimurium GN=CY43_22240 PE=4 SV=1 - [A0A023SY02_SALTM]	132.3312
6	F2FBE7	Phase-1 flagellin OS=Salmonella enterica subsp. enterica serovar Dublin str. SD3246 GN=SD3246_1252 PE=4 SV=1 - [F2FBE7_SALDU]	130.9255
7	F2FIW3	Putative Ig domain protein OS=Salmonella enterica subsp. enterica serovar Dublin str. SD3246 GN=SD3246_4515 PE=4 SV=1 - [F2FIW3_SALDU]	117.6328
8	A0A038EFQ2	Capsid protein OS=Salmonella enterica subsp. enterica serovar Typhimurium str. 108402 GN=N936_24625 PE=4 SV=1 - [A0A038EFQ2_SALTM]	73.45094
9	P0A1D3	60 kDa chaperonin OS=Salmonella typhimurium (strain LT2 / SGSC1412 / ATCC 700720) GN=groL PE=3 SV=2 - [CH60_SALTY]	55.89346
10	H8M1H4	Phosphoglycerate kinase OS=Salmonella enterica subsp. enterica serovar Typhimurium str. 798 GN=pgk PE=3 SV=1 - [H8M1H4_SALTM]	52.7316
